# Ketogenic diets in clinical psychology: examining the evidence and implications for practice

**DOI:** 10.3389/fpsyg.2024.1468894

**Published:** 2024-09-26

**Authors:** Nicole Laurent, Erin L. Bellamy, Donika Hristova, Ally Houston

**Affiliations:** ^1^Family Renewal, Inc., Vancouver, WA, United States; ^2^University of East London, London, United Kingdom; ^3^Transformation Evoked, Reston, VA, United States; ^4^University of Oxford, Oxford, United Kingdom

**Keywords:** psychology, clinical psychology, ketogenic diet, ketogenic metabolic therapy, metabolic psychiatry, bipolar disorder, schizophrenia, depression

## Abstract

**Introduction:**

The application of ketogenic dietary interventions to mental health treatments is increasingly acknowledged within medical and psychiatric fields, yet its exploration in clinical psychology remains limited. This article discusses the potential implications of ketogenic diets, traditionally utilized for neurological disorders, within broader mental health practices.

**Methods:**

This article presents a perspective based on existing ketogenic diet research on historical use, biological mechanisms, and therapeutic benefits. It examines the potential application of these diets in mental health treatment and their relevance to clinical psychology research and practice.

**Results:**

The review informs psychologists of the therapeutic benefits of ketogenic diets and introduces to the psychology literature the underlying biological mechanisms involved, such as modulation of neurotransmitters, reduction of inflammation, and stabilization of brain energy metabolism, demonstrating their potential relevance to biopsychosocial practice in clinical psychology.

**Conclusion:**

By considering metabolic therapies, clinical psychologists can broaden their scope of biopsychosocial clinical psychology practice. This integration provides a care model that incorporates knowledge of the ketogenic diet as a treatment option in psychiatric care. The article emphasizes the need for further research and training for clinical psychologists to support the effective implementation of this metabolic psychiatry intervention.

## 1 Introduction

Most first-line treatments for psychiatric disorders consist of psychotherapy and pharmacology. Psychotherapy exhibits positive effects independently of pharmacological treatments (Cuijpers et al., 2023) but often individuals are prescribed a combination of treatments.

Although psychotropic medications have increased substantially in recent decades (Brauer et al., 2021), the global burden of mental illness is rising across diverse populations (GBD 2019 Mental Disorders Collaborators, 2022). Given the persistent rise in mental illness despite advancements in psychotherapy and pharmacology, there is a pressing need for innovative, low-risk, and practical interventions that enhance the efficacy of psychotherapy.

This article intends to inform psychologists about the use of ketogenic diets as a treatment for mental illness and provide a better understanding of how their use in psychiatry informs the biopsychosocial model of practice. It introduces ketogenic diets, detailing their historical use for neurological disorders, relevant underlying biological mechanisms, their potential application in mental health treatment, and the relevance of ketogenic diets to clinical psychology research and practice.

According to the research, fasting has been used as a treatment for epilepsy since the Hippocratic era (Barzegar et al., 2021; Ferraris et al., 2023). In 1911 the first research study on fasting in epilepsy was published (Guelpa, 1911). As fasting could not be maintained indefinitely, Wilder et al. created a fasting-mimicking diet, by increasing ketones and called it the ‘Classic’ ketogenic diet because of its ketone-producing effects (Wilder, 1921; Kim, 2017). This was a significant medical breakthrough at a time when few treatments for epilepsy existed. This fasting-mimicking ketogenic diet has been studied for over 100 years for the treatment of neurological disease, particularly treatment-resistant strains of epilepsy in children (Martin-McGill et al., 2020). In the literature, there are compelling reasons to suggest that a therapeutic ketogenic diet may be effective in treating other neurological and psychiatric disorders (Kraeuter et al., 2020). Some case series and small studies have shown early promise in this supposition, including major depressive disorder (MDD), schizophrenia (Sethi et al., 2024), and bipolar disorder (BPD) (Danan et al., 2022; Sethi and Ford, 2022).

So far, there has been limited integration of dietary interventions in the clinical setting, apart from the practice of dieticians and nutritionists, although there have been calls to do so (La Puma, 2016; Unwin et al., 2022). Given the prominent role played by clinical psychologists in mental health diagnosis and treatment, there is potential for them to incorporate into their practice the support of valid and effective dietary interventions adjunct to their therapies and treatments. Specialized training for clinical psychologists in supporting therapeutic ketogenic dietary interventions may empower the successful backing of such interventions, which could lead to improved mental health outcomes for individuals with mental illness.

In addition to ketogenic metabolic therapy, psychologists are also encouraged to expand their knowledge and professional application to include other metabolic interventions. These interventions, like ketogenic diet implementation, often involve multi-step, individualized processes that require adaptation to the unique needs and circumstances of each patient. A considerable number of patients seen by general practitioners suffer from stress-related conditions, anxiety, depression, sleep issues, somatoform disorders, addictions, and metabolic problems. Unfortunately, many of these individuals do not receive adequate psychiatric care, representing a critical public health issue. However, those who are hesitant to pursue psychiatric treatment may still benefit significantly from receiving medical and psychological support for metabolic interventions. Psychologists’ understanding and support of ketogenic diets as a treatment for mental illness could enhance patient care in primary settings by providing additional therapeutic options for those not receiving specialized psychiatric services.

The relevance of extending knowledge of ketogenic diets to clinical psychologists lies in their biopsychosocial model of practice. Clinical psychologists are trained to utilize this biological pillar of practice to inform their work with patients, and the research evidence regarding therapeutic ketogenic diets as a treatment for neurological disorders and mental illness has reached a point in which this must be considered for adoption into informed psychological practice.

A therapeutic ketogenic diet, also known as ketogenic therapy, is a specific dietary regimen with a distinctive macronutrient distribution featuring high fat, moderate protein, and low carbohydrate levels (Ferraris et al., 2023). Carbohydrates are a nonessential macronutrient in the body, as the liver can create its own glucose on demand, so there is no risk of carbohydrate deficiency (Bier et al., 1999). The diet’s main mechanism of action is switching from the body’s primary fuel source, glucose via carbohydrates, to fat. This way of eating has been shown to mimic the effects of fasting by achieving a natural and safe metabolic state called “ketosis” (Kim, 2017). Ketosis is a process that converts dietary fat or liberated fat stores into ketone bodies in the liver which then supplies energy to the body and brain. For this reason, the ketogenic diet is often discussed informally as a metabolic psychiatry intervention. By adjusting brain metabolism, the diet not only addresses symptoms but potentially alters the underlying metabolic disturbances associated with various mental health disorders (Sethi and Ford, 2022).

Often, concerns around the safety of the ketogenic diet and the potential for negative side effects have been raised (McDonald and Cervenka, 2018). Though the ketogenic diet is considered a safe treatment for pediatric epilepsy (Faheem et al., 2024), various adverse effects have been reported in the research literature (Patikorn et al., 2023). However, side effects are often preventable and treatable if the diet is implemented correctly (Wells et al., 2020).

## 2 Treatment effects and mechanisms of action

Many mechanisms have been proposed to ensure the efficacy of a ketogenic diet in neurological and psychiatric disorders. Several relevant pathological states, for example, cerebral glucose hypometabolism, insulin resistance, neurotransmitter imbalances, mitochondrial dysfunction, oxidative stress, and inflammation, may be improved by following a ketogenic diet. Table 1 provides an overview of literature published showing relevant states of pathology seen in certain mental illnesses that are modifiable by ketogenic diets. Ketogenic diets have been shown to improve mitochondrial energy metabolism, inflammatory processes, oxidative stress, monoaminergic activity, and neuro-degeneration (Norwitz et al., 2020).

**Table 1:** Established ketogenic diet effects on pathological mechanisms in mental illness.

Diagnosis	Brain glucose hypometabolism	Neuro inflammation	Neurotransmitter imbalance	Oxidative stress
Schizophrenia	Pillinger et al. (2017) and Townsend et al. (2023)	Pasternak et al. (2016)	Bansal and Chatterjee (2021)	Bitanihirwe and Woo (2011) and Więdłocha et al. (2023)
Bipolar disorder	Ketter et al. (2001), Hosokawa et al. (2009), Wu et al. (2021), and Campbell and Campbell (2024)	Rao et al. (2010), Liu et al. (2018), Benedetti et al. (2020), Jones et al. (2021), and Lima et al. (2022)	Liu et al. (2018) and Martino and Magioncalda (2022)	Scaini et al. (2016), Kim et al. (2017), Steullet et al. (2018), Knorr et al. (2019), and Jiménez-Fernández et al. (2021)
Major depressive disorder	Su et al. (2014)	Goldsmith et al. (2016), Lamers et al. (2019), Osimo et al. (2019), and Bertollo et al. (2024)	Duman et al. (2019) and Sarawagi et al. (2021)	Ait Tayeb et al. (2023) and Liu et al. (2024)
Obsessive compulsive disorder	Hou et al. (2022)	Gerentes et al. (2019)	Ting and Feng (2008)	Kar and Choudhury (2016)
Generalized anxiety disorder	Blázquez et al. (2022)	Costello et al. (2019), Won and Kim (2020), and Guo et al. (2023)	Gkintoni and Ortiz (2023) and Mishra and Varma (2023)	Fedoce et al. (2018) and Blázquez et al. (2022)
Relevant ketogenic diet effects	Campbell and Campbell (2024)	Koh et al. (2020) and Jiang et al. (2022)	Sethi and Ford (2022)	Yu et al. (2023)

A ketogenic diet may also improve cognitive function, in both working memory and speed of processing (Mohorko et al., 2019). Beyond comorbid metabolic disorders characterized by insulin resistance, MDD and BPD medications frequently negatively impact metabolism (Chokka et al., 2006; Gramaglia et al., 2018). The ketogenic diet has been shown to significantly improve metabolic syndrome and metabolism (Bhanpuri et al., 2018; Miller et al., 2018; Sethi et al., 2024). Therefore the ketogenic diet may serve to mitigate some negative side effects of medications and could offer additional benefits on pathology (Sethi et al., 2024).

Improvements from a ketogenic diet have also been observed in neurological illnesses such as traumatic brain injury (TBI) (Prins et al., 2005), autism spectrum disorder (ASD) (Evangeliou et al., 2003), Parkinson’s disease, and Alzheimer’s disease (Paoli et al., 2014), along with improved memory (Krikorian et al., 2012), increased cognitive performance (Xu et al., 2010) and protection against cognitive impairment (Davidson et al., 2013). The positive effects of the ketogenic diet for multiple sclerosis (Storoni and Plant, 2015) due to the diet’s immunomodulating effects have also been reported (Brenton et al., 2022). In one study, participants with Parkinson’s disease also found improvements in anxiety after 12 weeks of following a ketogenic diet with <16 g carbohydrates per day (Murphy et al., 2004).

While using the ketogenic diet to treat epilepsy, it was observed that attention and cognition improved, and the diet appeared to have mood stabilizing effects (Murphy et al., 2004; Brietzke et al., 2018; Campbell and Campbell, 2019; Norwitz et al., 2020). Closer investigations in some studies showed that the ketogenic diet exhibited potential mood stabilization effects through modifications in metabolite levels, such as regulation of neurotransmitters including GABA, glutamatergic neurotransmission, mitochondrial function, and oxidative stress in patients following the ketogenic diet (Kraeuter et al., 2020; Norwitz et al., 2020). Some studies have proposed that changes in brain energy metabolism and the increased utilization of ketones for energy influence the neurotransmitter levels (Norwitz et al., 2020; Myette-Côté et al., 2022).

Further, when ketones are present in the blood, a reduction in neuronal excitability and anticonvulsant effects have been observed (Masino and Rho, 2012; Sethi and Ford, 2022; Omori et al., 2023). The anticonvulsant drugs prescribed for epilepsy treatment (e.g., lithium) are also prescribed for mood stabilization in mood disorders such as bipolar disorder, suggesting that epilepsy and bipolar disorder share some aspects of their etiologies (Qaswal, 2020). A ketogenic diet elicits beneficial effects without needing to administer the anticonvulsant drug and, in some cases, can exceed the levels of mood stabilization achieved with medications (Phelps et al., 2013).

## 3 Ketogenic diet in the context of the biopsychosocial model

The role of brain metabolism in the etiology of mental illness has largely been an uncharted territory within the field of psychology, primarily addressed within psychiatric literature (Sarnyai and Palmer, 2020). This oversight has meant that psychologists, for the most part, may not be aware of the significance of metabolic factors in mental health conditions. The robust body of evidence emerging from psychiatric research, particularly concerning the effects of ketogenic diets as a metabolic therapy for mental illness, presents an opportunity for its integration into psychological practice, as summarized in Table 2, which outlines a sample of completed and ongoing research using ketogenic diets for psychiatric disorders.

**Table 2:** Sample of current research investigating ketogenic diet with specific DSM-V diagnoses.

Diagnosis	Published and ongoing studies on ketogenic diet efficacy		
	Case studies	Clinical trials	Clinical trials in progress
Schizophrenia	Kraft and Westman (2009), Palmer (2017), and Palmer et al. (2019)	Danan et al. (2022) and Sethi et al. (2024)	Longhitano et al. (2024), Ruusunen (2024), and Ford (2024)
Bipolar disorder	Phelps et al. (2013), Saraga et al. (2020), and Chmiel (2022)	Danan et al. (2022), Needham et al. (2023), and Sethi et al. (2024)	Liwinski (2023), Brietzke (2023), Chouinard (2024), Longhitano et al. (2024), Phillips (2024), and Ruusunen (2024)
Depression	Palmer (2017), Saraga et al. (2020), and Calabrese et al. (2024)	Danan et al. (2022)	Brietzke (2023), Liwinski (2023), Volek (2023), and Gao and Chang (2024)

This integration, as stated earlier, aligns with the biological pillar of the biopsychosocial model, enhancing the understanding of mental disorders from a metabolic perspective and offering a new dimension to psychological treatment approaches. Building on this enhanced metabolic understanding, psychologists are uniquely equipped to translate these biological insights into practical psychological interventions that support patient care.

The established research using a therapeutic ketogenic diet as a metabolic treatment for mental illness (Sethi and Ford, 2022; Mentzelou et al., 2023) has potential implications for clinical psychology. There is a growing interdisciplinary recognition of the interplay between metabolism and mental illness that challenges existing paradigms and necessitates a reevaluation of conventional treatment approaches in psychiatry (Omori et al., 2023). The field of clinical psychology benefits from this conversation, discussing the relevance of ketogenic diets as a treatment for mental illness, aligning with Sim’s broader advocacy for the inclusion of diverse, empirically supported treatments in clinical psychology, a principle that extends to innovative approaches like the ketogenic diet (Sim, 2006).

The adoption of ketogenic diet as a metabolic therapy in clinical psychology is grounded in scientific research and evidence. It represents a commitment to an evidence-based approach in the field, where interventions are chosen based on empirical support for their efficacy and safety. This approach does not challenge existing paradigms but offers an expanded understanding of the neuroendocrine and neurobiological mechanisms involved in mental disorders (Norwitz et al., 2020; Giel et al., 2022; Campbell and Campbell, 2023). Supplementary Table S1 provides an overview of potential competencies where psychological expertise may support the integration and implementation of ketogenic metabolic therapy in mental health practice. Psychologists, with their proficiency in supporting medical treatments like medication adherence using well-established psychosocial interventions (Depp et al., 2008), are well-equipped to extend support to metabolic therapies like the ketogenic diet.

Extending their role in medical treatment support, psychologists can be trained to incorporate support for the ketogenic diet as a metabolic therapy into their clinical practice. Their extensive training in psychological and social factors plays an important role in the effective implementation and integration of this therapy into the patients’ lives, providing essential support for patients engaging with the ketogenic diet as a therapeutic intervention. Support provided by psychologists may include psychoeducation, addressing misconceptions about the ketogenic diet, utilizing strategies to enhance adherence and collaboration with other healthcare professionals, and ensuring a comprehensive and multidisciplinary care plan for patients.

Psychological interventions can help patients use ketogenic diets as a metabolic therapy in their treatment of mental illness and are well-trained in providing behavioral and emotional support to individuals attempting to make changes in alignment with their mental health goals. Psychologists can use existing theoretical orientations and choose treatment targets to assist patients in the exploration and implementation of the ketogenic diet as a treatment option. Psychologists who provide support to patients attempting to use ketogenic diets already benefit from the long-standing use of models traditionally used to support medication compliance in patients working with prescribers. Their role as important members of multi-disciplinary teams is well-established (Bisbey et al., 2019; Proctor and Vu, 2019; Wood et al., 2019) and may benefit research and treatment teams that consist of medical professionals such as psychiatrists, dieticians, or nutritionists.

Psychologists’ deep understanding of social and cultural influences plays a pivotal role in guiding patients through the adoption of ketogenic diets, and their expertise enables them to facilitate patient exploration of how societal norms and individual dietary choices impact the successful integration of this therapy. This allows psychologists to implement an approach that is essential for a comprehensive understanding of mental health care, bridging individual behavioral changes with broader social and cultural contexts. Recognizing the intricate interplay between societal norms, individual dietary choices, and mental health care, psychologists are uniquely positioned to understand and navigate the diverse cultural and social factors that influence the adoption of ketogenic diets as a therapeutic intervention. Cultural traditions, personal beliefs, and existing social norms deeply affect individuals’ food choices, their relationship with food, and their sense of self. Food choices have deeply embedded cultural significance, playing roles in social gatherings, and can impact the adoption of ketogenic diets as a treatment modality for diverse populations. Psychologists have expertise in understanding these diverse influences and the ability to appreciate the complexity of making dietary choices within different cultural contexts, which can assist patients in being able to implement the therapy successfully while maintaining their relationships and cultural identity (Clauss-Ehlers et al., 2019).

Psychologists not only provide these potentially invaluable contributions in assisting patients using ketogenic diets as metabolic therapy for mental illness, but their contributions to the research literature in the form of quantitative and qualitative analysis can also help ensure improved treatment outcomes. This research is particularly important for tailoring the therapy to meet the unique needs of diverse populations, including those with dual diagnoses. Beyond patient support, psychologists’ insights are vital in research, potentially enhancing understanding of how cultural and social factors influence the effectiveness of ketogenic diets in mental health treatment and providing additional relevant data on treatment outcomes.

## 4 Conclusion

The inclusion of accurate knowledge of this intervention offers a promising complement to the existing array of evidence-based interventions in the biopsychosocial model of psychology practice, paving the way for advancements in mental health treatment. Such integration marks a meaningful broadening of clinical psychology’s scope that mirrors the profession’s commitment to stay abreast of and responsive to evolving scientific insights as part of competent psychological practice.

In their role as clinicians and researchers, psychologists are uniquely equipped to explore and support patient use of the ketogenic diet in mental health care. Their expertise in psychological assessment and intervention is critical for understanding and optimizing the use of this therapy in diverse patient populations. As the field continues to evolve, psychologists’ engagement with current research and clinical applications of the ketogenic diet as a therapeutic intervention will be instrumental in shaping effective, evidence-based mental health treatments.

## Data Availability

The original contributions presented in the study are included in the article/Supplementary material, further inquiries can be directed to the corresponding author.

## Supplementary material

Supplementary Table S1 Areas of psychological expertise relevant to ketogenic metabolic therapy or some variation?
