# Supplementary material for: Ketogenic diets in clinical psychology: examining the evidence and implications for practice
Source: Front Psychol. 2024 Sep 26;15:1468894. doi: 10.3389/fpsyg.2024.1468894 (PMC11464436; doi:10.3389/fpsyg.2024.1468894)
Supplement: Supplementary Table S1 — Areas of psychological expertise relevant to ketogenic metabolic therapy or some variation? [file Table_1.DOCX]

**Supplementary Table 1: Areas of Psychological Expertise Relevant to Ketogenic Metabolic Therapy**

| Competency | Relevant Expertise Domains | Examples of Potential Contribution |
| --- | --- | --- |
| Research and Development | Population-Specific Research | Investigating the effects of ketogenic metabolic therapy on diverse patient populations |
|  | Innovative Therapeutic Applications | Exploring the application of ketogenic therapy in various psychiatric conditions |
|  | Conducting Qualitative Research | Providing qualitative insights into patient experiences with ketogenic therapy |
|  | Development of Psychometric Instruments | Designing and validating tools relevant to ketogenic therapy implementation |
| Patient Support and Counseling | Psychotherapy Integration | Utilizing psychotherapy to support patients in implementing ketogenic therapy |
|  | Behavioral and Emotional Support | Assisting patients in managing cognitive, emotional, and social challenges related to ketogenic therapy |
|  | Motivation and Adherence Strategies | Developing behavioral strategies to enhance patient adherence to ketogenic therapy |
|  | Social and Cultural Adaptation | Supporting patients in adapting ketogenic therapy within their social and cultural contexts |
| Professional Collaboration | Interdisciplinary Collaboration | Collaborating with healthcare professionals (e.g., dieticians, physicians) in a multidisciplinary approach to patient care |
|  | Expanding Access and Practice | Exploring ways to increase access to ketogenic therapy in underserved populations |
|  | Ethical Practice and Scope Management | Ensuring that psychological support for ketogenic therapy adheres to ethical standards and professional scope |
|  | Education and Advocacy | Educating other healthcare providers and advocating for psychological aspects of ketogenic therapy |

This table outlines domains where psychological expertise can contribute to the effective implementation and support of ketogenic metabolic therapy in clinical settings. Each domain is accompanied by specific examples of potential contributions that would fall within the scope of clinical psychology.
